# A case report of cryoablation for chronic shoulder pain due to osteoarthritis

**DOI:** 10.1016/j.inpm.2022.100146

**Published:** 2022-09-15

**Authors:** Adam Rupp, Preeti Panchang, McCasey Smith

**Affiliations:** Department of Physical Medicine and Rehabilitation, University of Kansas Medical Center, Kansas City, KS, USA; Department of Physical Medicine and Rehabilitation, University of Kansas Medical Center, Kansas City, KS, USA; Department of Physical Medicine and Rehabilitation, University of Kansas Medical Center, Kansas City, KS, USA

Dear Editor,

Shoulder or glenohumeral osteoarthritis is a relatively common contributor of shoulder pain. Osteoarthritis (OA) is a complex multifactorial process that not only causes debilitating pain but can also impair one's function by reducing active and passive range of motion [1]. Management aims to improve both pain and function. Conservative treatments including over the counter medications, lifestyle changes and physical therapy have good efficacy in early stages but are limited in more advanced arthritis. Injections with steroids, long acting steroids (e.g. triamcinolone acetonide extended release) and platelet rich plasma can provide temporary relief and improve participation in therapies [2]. However formal guidelines for injections are limited given the variability in outcomes and the lack of evidence for any specific injectate type [[3], [4], [5]]. Lastly, surgical options usually involve mechanical replacement of the joint. Functional outcomes with surgery have improved over the years due to advancements in surgical technique and prosthesis design [6]. Despite this however, due to age, comorbidities, financial constraints or personal preferences surgery may not be an option. Thus, it is imperative to find alternative methods of management for these patients.

Cryoablation is an old method of managing pain that has regained popularity given advancements in technology, imaging and technique. Cryoablation is a procedure used to help manage persistent pain that involves freezing neural tissue to denature the nerve with a goal of achieving pain relief that lasts for several weeks to months. This technique has been used for decades in the management of chronic pain in various anatomical locations including knees and spinal facet joints. This was typically done in a controlled setting such as a fluoroscopy procedure suit [7]. However, recent advancements in ultrasound and the development of portable cryoablation devices has afforded physicians the ability to provide long lasting pain relief for chronic and post-surgical pain in an outpatient clinic setting [8].

The procedure itself involves reversibly ablating with extremely cold temperatures while providing anesthesia to a given area [8]. The cryoprobe consists of three telescoping tubes that act as conduits for gas and heat exchange. Freezing works as the gas (typically N2O or CO2) travels from the proximal high-pressure end to the distal low-pressure end of the tube, then rapidly expands leading to heat extraction. Temperatures can drop below −170 ​°C. The cooled gas is quickly vented back into the probe to prevent diffusion into the patient's tissues [9]. The probe is usually guided to the target nerve with ultrasound, fluoroscopy or computed tomography. A typical freeze cycle lasts 3–5 minutes. Once the freezing process has begun an “ice ball” is visualized on imaging allowing for direct monitoring of the locally affected structures. The ice ball typically reaches sizes of 3.5–5.5mm. A thawing period of usual 2 minutes follows freezing, this allows the ice ball to reduce in size prior to retraction [10].

Cryoablation differs from traditional ablative techniques such as radiofrequency neurotomy due to its preservation qualities. Cryoablation works by disrupting axonal continuity causing Wallerian degeneration while leaving the myelin, endoneurium, perineurium and epineurium intact. Preservation of this scaffolding allows for neuronal regeneration with the correct trajectory which not only lowers the incidence of neuritis or neuroma formation [9] but also contributes to the reversibility and preservation of the overall structure and function of the nerve [[11], [12], [13]].

Cryoablation is typically well tolerated with limited documented side effects such as depigmentation, cutaneous lesioning (frost bite) and ablating the wrong structure. Contraindications to percutaneous cryoablation include coagulopathy, cold urticaria, cryoglobulinemia and Raynauds. Cryoablation has been utilized for various pain syndromes including craniofacial pain, persistent pain from rib fractures, herpetic neuralgia in the thoracic region as well as lower back, knee and abdominal pain [8]. These typical utilities arise from the ability to identify and correctly target specific nerves.

This case study describes an additional utility for ultrasound guided cryoablation for chronic left osteoarthritic shoulder pain by targeting the suprascapular and Axillary nerves. The suprascapular nerve arises from the upper trunk of the brachial plexus and travels inferolateral to innervate the supraspinatus and then pass inferiorly through the suprascapular notch before diving into the infraspinatus. It also provides shared nerve innervation to the shoulder joint [7]. The Axillary nerve is the second nerve that provides innervation to the shoulder joint. The Axillary nerve arises distally from the posterior chord of the brachial plexus and traverses inferior and posteriorly through the quadrangular space with the posterior circumflex artery to innervate the deltoid, teres minor, triceps and provide cutaneous sensation to the lateral upper arm [14]. This case report presents on a patient who received cryoablation to the suprascapular and Axillary nerves for chronic left shoulder osteoarthritis pain.

Verbal consent was obtained from the patient to present and publish this case report. The patient is a 92-year-old female who initially presented to clinic with chronic severe left shoulder pain and limited range of motion. She rated her average pain at a VAS of 9/10. Initial physical exam was notable for crepitus as well as tenderness to the glenohumeral joint with limited abduction and external rotation. Left shoulder X-ray from 1-year prior showed moderate degeneration of the glenohumeral joint. Initial plan included serial anatomically guided glenohumeral joint steroid injections. She underwent three injections. The first two gave her 80% relief for 6 months, the third gave her less than 2 days of 50% relief. It was decided to trial extended release steroid injections (triamcinolone acetonide extended release) under ultrasound guidance to improve accuracy. She underwent another series of three injections lasting 2–3 months each, however she noted decreasing efficacy with each injection. At this point surgical evaluation was discussed however the patient declined and asked for alternative options. Her VAS at the time was 10/10. The possibility of percutaneous cryoneurolysis to the suprascapular nerve was discussed. The patient indicated that her main concern was shoulder pain and that she will consent both verbally and in a written form to move forward with the procedure.

For the procedure the patient was positioned in the seated position with her left arm draped over her lap. A linear ultrasound probe was used to provide direct visualization of the suprascapular nerve as it passes through the suprascapular notch. The area just lateral to the probe was marked and prepped in a sterile fashion. A 25-gauge 2-inch needle was inserted from lateral to medial and 2.5 mL of lidocaine 1%/Epinephrine (1:100,000) was injected, anesthetizing the area. The ultrasound probe was switched to curvilinear for better visualization. The cryoprobe was inserted, visualized and advanced in long axis view to the target nerve. 3 cycles of freezing to −70 ​°C with 2 minutes of thawing between each were completed. The patient tolerated the procedure well with no immediate complications. Four days after the treatment she had already regained the ability to lift her arm higher than she had been previously without pain. 1-month post-procedure she continued to have greatly overall reduced pain however she was still having some residual axillary region pain. It was decided to repeat cryoneurolysis to target the axillary nerve. 2 months after the initial treatment the patient again provided consent to move forward with the ablation.

Patient was positioned in the prone position. Similarly, to the initial procedure the axillary nerve was directly visualized utilizing a linear ultrasound probe. The nerve was seen adjacent to the circumflex artery just lateral to teres minor. The same methodology as the initial procedure was employed for the second treatment. Again, the patient tolerated the procedure well with no immediate complications. She was seen 1 month later and noted significant pain relief with a decrease in her VAS to 3/10. She also reported improved functional use of the hand and arm and was pleased with the results. No adverse effects from the procedure were reported.

The patient had the cryoablation procedure repeated two more times at 4-month intervals. She noted consistently 3.5 months of both pain and functional improvement. Overall, she has ongoing satisfaction with the procedure and stated she will continue to repeat this as able.

Cryoablation has historically been utilized for various pain syndromes including craniofacial pain, persistent pain from rib fractures, herpetic neuralgia in the thoracic region, lower back pain and abdominal pain [7]. The shoulder joint however, has not been well studied and to date there are limited case reports documenting cryoablation for this region. The above case report represents the first to document cryoablation for chronic shoulder osteoarthritis pain. Prior studies focused on other pain etiologies such as post-op rotator cuff surgery pain. One such case report done by Ilfeld et al. included 5 total patients who underwent preoperative ultrasound guided cryoneurolysis preceding rotator cuff repair (n ​= ​2) and total knee arthroplasty (TKA) (n ​= ​3). For the shoulder they targeted the suprascapular nerve near the suprascapular notch. They utilized a 5.5-cm, 22-G, single-use cryoneurolysis probe and performed two cycles of 3-min freezing to −70 ​°C, separated by a 2 minute defrost period. All five patients had pain scores under 2 post operatively and required less opioids then historical controls. Additionally, the rotator cuff patients achieved full range of motion 2–3 weeks after the procedure. No discomfort during or adverse effects after the procedure were reported [8].

Such as with Ilfeld's above study, more commonly is the use of cryoneurolysis for the knee and facet joints. A retrospective study by Urban et al. assessed a total of 267 patients after TKA, of which 169 had undergone cryoneurolysis prior to surgery. They collected primary outcomes looking at opioid usage at various points until 6 weeks post-op, in addition to several secondary outcome measurements. The cryoneurolysis group had an overall reduction in length of stay by about 44%, decreased opioid use during hospitalization as well as increase range of motion at discharge. Though lack of randomization, this study did show that when added to a multimodal pain protocol, post-TKA cryoneurolysis can assist with pain control, functional gain and the recovery process [15]. Another randomized control trial conducted by Radnovich et al. treated the infrapatellar branch of the saphenous nerve for patients with knee osteoarthritis. The study showed sustained improvement in the Western Ontario and McMaster University Osteoarthritis Index score of the study group after one session compared to the sham group. Improvements were noted for 90 days, with some noting statistically significant improvement at day 150. Some caution is indicated as blinding was not complete in the study [16].

There have also been multiple studies showing efficacy for lumbar and cervical facet joints. One such retrospective observational study conducted by Wolter et al. included 91 patient who underwent cryoneurolysis of the lumbar facet joints under CT guidance. At 18 months they found a VAS decrease from 7.7 to 3.72 (p ​< ​0.0001). Various functional improvement measures as well reached statistical significance [17]. A second study completed by Birkenmaier et all investigated 50 patients who underwent lumbar facet cryoablation. Similar to Wolter's study above, at 1 year follow up mean VAS decreased from 7.7 to 4.2 (p ​< ​0.0001) [18].

Though currently limited, the evidence does continue to grow regarding the safety, efficacy and utility of cryoablation, specifically for glenohumeral osteoarthritis. Improving the quality of evidence for these non-surgical treatments is imperative given the aging population. Other future possible glenohumeral osteoarthritis management modalities include peripheral nerve stimulation and pulsed radiofrequency ablation. Mansfield described a case report of a 91-year-old man with refractory advanced glenohumeral OA treated with a peripheral nerve stimulator. The stimulator was placed next to the axillary nerve in the quadrangular space. He reported 70% pain relief for more than 8 months [19,20]. A second innovative method of managing refractory OA shoulder pain is through pulsed radiofrequency ablation (RFA). Pulsed RFA is accomplished by applying short high-voltage bursts of current then allowing heat diffusion with longer, silent phases. This ultimately leads to reversible cell damage, causing nerve fibers to ironically “freeze”. This process potentially mimics that of cryoablation [21]. This process has been demonstrated in chronic shoulder pain due to rotator cuff pathologies and hemiplegic shoulder pain with good efficacy [[22], [23], [24]]. OA reports are limited but Pinto did conduct a prospective study evaluating 34 patients with arthrosis undergoing pulsed RFA. They found improvements in both pain and range of motion at 6 months post-procedure [25].

This case illustrates a potential use of cryoneurolysis to the suprascapular and axillary nerves for the treatment of chronic glenohumeral osteoarthritis pain in whom surgical treatment is not an option or preference. Further larger and higher quality studies utilizing cryoneurolysis for shoulder osteoarthritis are necessary before formal recommendations can be made.

## Financial disclosures

None.

## Declaration of competing interest

The authors declare that they have no known competing financial interests or personal relationships that could have appeared to influence the work reported in this paper.
